# Pharmacy—Full Text of the Law on That Subject

**Published:** 1889-06

**Authors:** 


### PHARMACY—FULL TEXT OF THE LAW ON THAT SUBJECT.

Following is the full text of the new pharmacy law, passed by the Twenty-first Legislature, with the exception of the emergency clause, which put the law into immediate effect:

Section 1—Be it enacted by the Legislature of the State of Texas: That it shall be unlawful for any person, unless a qualified pharmacist within the meaning of this act, to open or conduct any pharmacy or store for compounding medicines, or for any one not a qualified pharmacist, to prepare physicians' prescriptions, or compound medicines, except under the direct supervision of a qualified pharmacist, as hereinafter provided.

Section 2—Any person, in order to be qualified, shall be 21 years old and shall have passed a satisfactory examination before the Board of Pharmacy of Texas, or shall be a graduate in pharmacy or an assistant in pharmacy.

Section 3—Graduates in phaimacy shall be such as have obtained a diploma from a regular incorporated college of pharmacy, and that requires not less than two years' experience in stores where prescriptions of medical practitioners have been compounded before said diploma is issued.

Section 4—Assistants in pharmacy must be 21 years old and have had two years' experience in stores where prescriptions of medical practitioners have been prepared, and shall have passed a satisfactory examination before the Board of Pharmacy of Texas.

Section 5—As soon as convenient after the passage of this act, the presiding judge of the district court of the several judicial districts, shall, as soon as practicable, severally appoint a Board of Pharmaceutical Examiners for their respective districts, who shall hold their office two years, which appointment shall be in writing and signed by the judge making the same and delivered to the person appointed. Said Board of Pharmaceutical Examiners shall be composed of not less than three qualified pharmacists who are residents of the districts of which they are appointed. If a vacancy occurs in said board another shall be appointed, as aforesaid, to fill the unerpired term. Said board shall have power to make by-laws and all necessary regulations for the proper fulfillment of their duties under this act.

Section 6—The board shall meet within ninety days after the passage of this act, and once a year thereafter, in as central portions of the district as practicable, and shall give one month's notice through the public press of the time and place of such meeting. The board shall organize for business by electing a registrar of pharmacy. The duties of said board shall be to examine all applicants for registration; to direct the registration by the registrar of all persons properly qualified or entitled thereto.

Section 7—The duties of the registrar of pharmacy shall be to keep a book in which shall be entered, under the supervision of the Board of Pharmacy, the name and place of business of every person who shall apply for registration, and a statement, signed by the person making the application, of such facts in the case as may claim to justify his or her application. It shall also be the duty of the registrar to duly note the fact against the name of any qualified pharmacist who may have died or removed from the State, or disposed of or relinquished his business.

Section 8—Any person, in order to become a qualified pharmacist within the meaning of this act, shall apply and appear for examinnation and registration, and shall pay to the Board of Pharmacy $5; and on passing the examination required, shall be furnished free of cost a certificate of registration, signed by the said board. Should said person fail to pass a satisfactory examination, he may, at another one meeting of the Board of Pharmacy, within twelve months, be permitted to be examined without cost.

Section 9—Graduates, as specified in section 3, shall apply for registration and if they produce satisfactory evidence to the Board of Pharmacy that they have a right to be registered, shall, upon paying the said Board $3, be furnished a certificate of registration without examination.

Section 10—Proprietors who are actively engaged in the preparation of physicians' prescriptions and compounding and vending medicine in the State of Texas at the passage of this act, shall be exempt from examination; also assistants who are likewise engaged and have been so engaged for three years, and are 21 years old ; provided, he, she, or they will register as specified in this act at first meeting of the Board of Pharmacy, and upon paying the Board $3, shall be furnished with a certificate of registration; provided, that the provisions of this bill shall not prevent any person from engaging in the business herein described as proprietors or owners thereof; provided, such proprietor or owners shall have employed in his business some qualified pharmacist to fill prescriptions and compound drugs.

Section 11—All persons receiving a certificate of registration shall place it in a conspicuous place in their place of business. In failing to do this, the Board of Pharmacy shall cancel their registration and deprive them of their certificate.

Section 12—Any person not a qualified pharmacist, but continues to compound prescriptions or retail medicines without complying with this act, shall upon the first conviction be sentenced to pay a fine of not less than $50 nor more than $100; and upon the second and every subsequent conviction, shall be sentenced to a fine of not not less than $100 nor more than $200.

Section 13—Any person who shall procure or attempt to procure registration for himself or for another, under this act, by making or causing to be made any false representation, shall be deemed guilty of a misdemeanor, and shall be fined not less than $25 nor more than $100, and the name of the person so fraudulently registered shall be stricken from the register.

Section 14—Any member of the Board of Pharmacy may issue temporary certificates upon satisfactory proof that the applicant is competent; but said temporary certificate shall be null and void after the first regular or extra meeting of the Board next after granting said temporary certificate; provided further, that not more than one temporary certificate shall ever be granted to any one person.

Section 15—All courts having jurisdiction in criminal causes are required to give this act in charge to each grand jury impanelled in such courts.

Section 16– This act shall not apply to towns and cities containing less than 1,000 inhabitants. Towns and cities that arrive at one or more thousand inhabitants on and after the passage of this act shall come within its provisions. The manner of ascertaining the census shall be the last official one, whether it be federal, state, town, or city.

Section 17—Nothing in this act shall be construed to apply to any practitioner of medicine who does not keep open shop for compounding, dispensing, and selling medicines, nor so construed as to prevent any person or persons from investing their means in a drug store or stores; provided, they keep employed qualified pharmacists for the direct supervision of vending and compounding medicines.

---

RECEIVED of Drs. W. A. Morris, President, and F. E. Daniel, Secretary P. M. B. A., seventy-nine dollars collected under assessment for Dr. W. F. Buck's beneficiary.

MISS MINNIE BUCK,
per J. B. Earl.

Other receipts and names of payees will appear in July number.

